# CSF synaptic protein concentrations are raised in those with atypical Alzheimer’s disease but not frontotemporal dementia

**DOI:** 10.1186/s13195-019-0564-2

**Published:** 2019-12-17

**Authors:** Mica T. M. Clarke, Ann Brinkmalm, Martha S. Foiani, Ione O. C. Woollacott, Carolin Heller, Amanda Heslegrave, Ashvini Keshavan, Nick C. Fox, Jonathan M. Schott, Jason D. Warren, Kaj Blennow, Henrik Zetterberg, Jonathan D. Rohrer

**Affiliations:** 1 Dementia Research Centre, Department of Neurodegenerative Disease, University College London, Queen Square Institute of Neurology, London, WC1N 3BG UK; 2 Department of Psychiatry and Neurochemistry, Institute of Neuroscience and Physiology, The Sahlgrenska Academy at the University of Gothenburg, Mölndal, Sweden; 3 UK Dementia Research Institute at UCL, London, UK; 4 Department of Neurodegenerative Disease, University College London, London, UK; 5 Clinical Neurochemistry Laboratory, Sahlgrenska University Hospital, Mölndal, Sweden

**Keywords:** Alzheimer’s disease, Frontotemporal dementia, Synaptic, Biomarkers, Neurogranin, SNAP-25, Synaptotagmin-1

## Abstract

**Background:**

Increased CSF levels of a number of synaptic markers have been reported in Alzheimer’s disease (AD), but little is known about their concentrations in frontotemporal dementia (FTD). We investigated this in three synaptic proteins, neurogranin, SNAP-25, and synaptotagmin-1.

**Methods:**

CSF samples were analysed from 66 patients with a disorder in the FTD spectrum and 19 healthy controls. Patients were stratified by their tau to Aβ_42_ ratio: those with a ratio of > 1 considered as having likely AD pathology, i.e. an atypical form of AD (‘AD biomarker’ group [*n* = 18]), and < 1 as likely FTD pathology (‘FTD biomarker’ group [*n* = 48]). A subgroup analysis compared those in the FTD group with likely tau (*n* = 7) and TDP-43 (*n* = 18) pathology. Concentrations of neurogranin were measured using two different ELISAs (Ng22 and Ng36), and concentrations of two SNAP-25 fragments (SNAP-25tot and SNAP-25aa40) and synaptotagmin-1 were measured via mass spectrometry.

**Results:**

The AD biomarker group had significantly higher concentrations of all synaptic proteins compared to controls except for synaptotagmin-1 where there was only a trend to increased levels—Ng22, AD mean 232.2 (standard deviation 138.9) pg/ml, controls 137.6 (95.9); Ng36, 225.5 (148.8) pg/ml, 130.0 (80.9); SNAP-25tot, 71.4 (27.9) pM, 53.5 (11.7); SNAP-25aa40, 14.0 (6.3), 7.9 (2.3) pM; and synaptotagmin-1, 287.7 (156.0) pM, 238.3 (71.4). All synaptic measures were significantly higher in the atypical AD group than the FTD biomarker group except for Ng36 where there was only a trend to increased levels—Ng22, 114.0 (117.5); Ng36, 171.1 (75.2); SNAP-25tot, 49.2 (16.7); SNAP-25aa40, 8.2 (3.4); and synaptotagmin-1, 197.1 (78.9). No markers were higher in the FTD biomarker group than controls. No significant differences were seen in the subgroup analysis, but there was a trend to increased levels in those with likely tau pathology.

**Conclusions:**

No CSF synaptic proteins have been shown to be abnormal in those with likely FTD pathologically. Higher CSF synaptic protein concentrations of neurogranin, SNAP-25, and synaptotagmin-1 appear to be related to AD pathology.

## Background

Frontotemporal dementia (FTD) is a clinically, pathologically, and genetically heterogeneous disorder with few biomarkers that can currently either detect neurodegeneration in the early stages of disease or track disease progression [1]. Synapse dysfunction occurs early in the pathogenesis of neurodegenerative diseases, leading to degradation of vital connections within neuronal networks. Biomarkers of synaptic integrity may therefore represent a potentially more sensitive way of measuring disease onset and severity [2–4].

Most work so far in synaptic biomarkers has focused on cerebrospinal fluid (CSF) where increased protein concentrations are thought to reflect degeneration of functional synapses [2]. In Alzheimer’s disease (AD), the concentration of the postsynaptic protein neurogranin, which is involved in synaptic plasticity and enhances the strength of connections, is elevated [2, 5–9], and in amyloid-positive people with mild cognitive impairment, raised neurogranin levels are associated with cognitive decline and conversion to dementia [10]. Only a few studies have investigated other dementias, with concentrations in FTD reported as lower than AD [6, 8, 11, 12].

Less research has been performed in the measurement of other synaptic proteins in CSF. Synaptosomal-associated protein 25 (SNAP-25) is critical for synaptic vesicle fusion to the membrane, and its concentration has been found to be significantly increased in CSF of those with AD compared to controls [13]. Similarly, CSF concentration of the presynaptic vesicle protein synaptotagmin-1, essential for exocytosis of synaptic vesicles and neurotransmitter release, has also been shown to be significantly increased in people with AD compared to controls [14]. Neither SNAP-25 nor synaptotagmin-1 has been previously investigated in people with FTD.

In this study, we used immunochemical and mass spectrometric methods to measure neurogranin, SNAP-25, and synaptotagmin-1 in CSF to determine whether these synaptic proteins were specific to those with AD pathologically, or whether they were also abnormal in those with likely FTD-related pathology.

## Methods

### Participants

Sixty-six participants with a diagnosis in the FTD and primary progressive aphasia spectrum were consecutively recruited to the University College London FTD cohort studies. These included 21 patients with behavioural variant FTD (bvFTD) [15] and 45 with primary progressive aphasia (PPA) [16]: 11 with semantic variant PPA (svPPA), 16 with non-fluent variant PPA (nfvPPA, 2 of whom had concomitant progressive supranuclear palsy, PSP), 15 with logopenic variant PPA (lvPPA), and 3 with PPA not meeting criteria for a specific variant (here called PPA-not otherwise specified, PPA-NOS). All participants underwent genetic screening, which revealed pathogenic mutations in 10 patients: 4 *MAPT*, 3 *GRN*, and 3 *C9orf72* mutations. Nineteen healthy control participants were also recruited. The study was approved by the local ethics committee, and all participants consented to take part.

### Measurement of CSF proteins

All CSF was collected, processed, and stored at − 80 °C following standardised procedures. Measurement of CSF proteins occurred after all participants had been recruited. The protein concentrations of established biomarkers of CSF total-tau and Aβ_42_ were measured using sandwich enzyme-linked immunosorbent assays (ELISAs; INNOTEST®, Fujirebio Europe N.V., Gent, Belgium) following the manufacturer’s instructions.

### Immunochemical assays

Two separate ELISAs were performed for the measurement of neurogranin (Ng) in CSF using in-house generated antibodies: either Ng22 or Ng36 (two different clones, both raised against amino acids 63-75 of Ng) as detector, and Ng2 (epitope 52-64) as capture antibody [12, 17]. We denote the two Ng measures as Ng22 and Ng36 on the basis of the detector antibody clone used.

### Mass spectrometry

High-resolution parallel reaction monitoring was performed on a Q Exactive quadropole-orbitrap mass spectrometer coupled to an Ultimate 3000 chromatography system (Thermo Fisher Scientific) for the parallel quantification of SNAP-25 (amino acids 32-40 = SNAP-25aa40 and Ac-2-16 = SNAP-25tot) and synaptotagmin-1 (amino acids 215-228) in CSF. Antibodies included mouse monoclonal antibody SP12 recognising SNAP-25 and mouse monoclonal antibody SM181 recognising an epitope containing the N-terminally acetylated first 11 amino acids of SNAP-25 (as previously described by Brinkmalm et al. [13]), and monoclonal antibody clone 41.1 recognising the cytoplasmic portion of synaptotagmin-1 from Synaptic Systems (detailed methods previously described by Öhrfelt et al. [14] and Fernström et al. [18]).

### Participant stratification

In the primary analysis, participants with dementia were stratified by their total-tau and Aβ_42_ concentrations into an ‘AD biomarker group’ (i.e. those likely to have AD pathologically (atypical AD); tau to Aβ_42_ ratio of > 1 [19]: *n* = 18, mean age at CSF sample collection 66.4 years (standard deviation 5.9), male to female ratio 11:7; 13 patients with lvPPA, 3 with nfvPPA, 1 with svPPA, and 1 with PPA-NOS) and an ‘FTD biomarker group’ (i.e. those likely to have frontotemporal lobar degeneration pathologically; tau to Aβ_42_ ratio of < 1: *n* = 48, age at CSF 64.0 (6.8), 34:14), and compared with healthy controls (all of whom had a tau to Aβ_42_ ratio of < 1) (Table 1).

**Table 1:** Baseline characteristics of the cohort

Participant stratification	Number	Age at CSF (mean (SD))	Gender (M:F)	CSF total-tau (pg/ml, mean (SD))	CSF Aβ_42_ (pg/ml, mean (SD))	Total-tau to Aβ_42_ ratio	Disease duration (mean (SD))	MMSE (mean (SD))
Control	19	64.2 (6.9)	10:9	327.9 (93.2)	1012.7 (237.3)	0.3 (0.1)	N/A	N/A
AD biomarker group	18	66.4 (5.9)	11:7	984.4 (543.6)	471.1 (179.0)	2.5 (2.0)	3.5 (2.0)	21.1 (5.2)
FTD biomarker group	48	64.0 (6.8)	34:14	376.1 (173.3)	851.2 (246.1)	0.5 (0.2)	5.7 (4.1)	23.9 (7.0)
Probable TDP-43 pathology	18	62.6 (5.6)	13:5	456.0 (226.0)	863.4 (222.5)	0.5 (0.3)	6.6 (4.9)	23.3 (6.7)
Probable tau pathology	7	64.7 (8.9)	5:2	444.4 (146.4)	894.3 (214.1)	0.5 (0.2)	5.5 (3.1)	21.1 (10.4)

*MMSE* Mini-Mental State Examination

In a secondary analysis, a subgroup of participants in the FTD biomarker group were stratified according to whether they were likely to have probable TDP-43 pathology (*n* = 18; clinical diagnosis of svPPA or FTD with motor neurone disease or genetic diagnosis of a *GRN* or *C9orf72* mutation: age at CSF 62.6 years (5.6), 13:5) or probable tau pathology (*n* = 7; clinical diagnosis of nfvPPA with PSP, genetic diagnosis of a *MAPT* mutation, and a pathological diagnosis of corticobasal degeneration: age at CSF 64.7 (8.9), 5:2), and compared with healthy controls. Twenty-three of the forty-eight participants in the FTD biomarker group could not be stratified into either group.

### Statistical analyses

All statistical analyses were performed using STATA (v.14). Linear regression analysis with 95% bias-corrected bootstrapped confidence intervals (CIs) with 1000 repetitions was used to compare concentrations of all synaptic proteins between groups. There was no difference in age between groups, but gender was significantly different between groups and was adjusted for in analyses. Spearman’s correlation coefficient was used to investigate the association between each synaptic protein concentration, and between synaptic protein concentrations and both CSF total-tau and Aβ_42_ concentrations.

### Data availability

Data from the study will be available on request.

## Results

### Biomarker group stratification

**Fig. 1:**
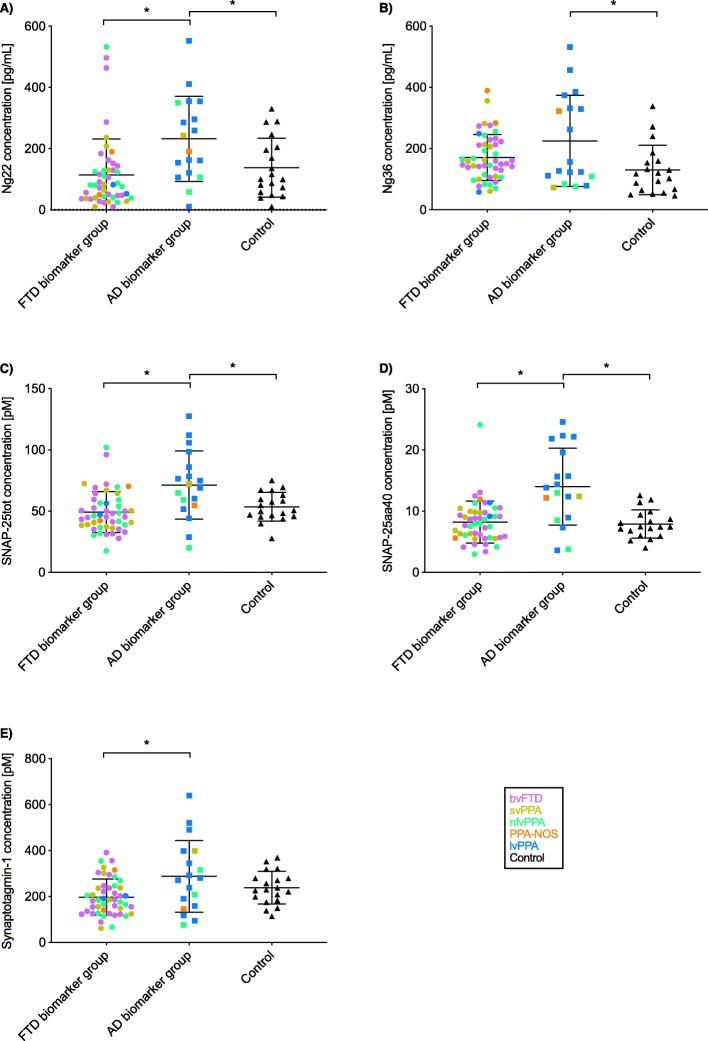
Concentrations for the five synaptic measures in the AD biomarker group, FTD biomarker group, and controls: **a** Ng22, **b** Ng36, **c** SNAP-25tot, **d** SNAP-25aa40, and **e** synaptotagmin-1. Within each group, data points are coloured to represent the clinical phenotype of each participant: purple = bvFTD, yellow = svPPA, green = nfvPPA, blue = lvPPA, orange = PPA-NOS, and black = controls

Concentrations of all synaptic markers were significantly increased in the AD biomarker group compared to the control group except for synaptotagmin-1 where there was only a trend to higher levels: mean (standard deviation) Ng22, 232.2 (138.9) vs 137.6 (95.9) pg/ml; Ng36, 225.5 (148.8) vs 130.0 (80.9) pg/ml; SNAP-25tot, 71.4 (27.9) vs 53.5 (11.7) pM; SNAP-25aa40, 14.0 (6.3) vs 7.9 (2.3) pM; and synaptotagmin-1, 287.7 (156.0) vs 238.3 (71.4) pM (Fig. 1, Tables 2 and 3).

**Table 2:** CSF protein concentrations of each synaptic measure for each biomarker group

Synaptic measure	FTD biomarker group (mean (SD))	AD biomarker group (mean (SD))	Control group (mean (SD))
Ng22 (pg/ml)	114.0 (117.5)	232.2 (138.9)	137.6 (95.9)
Ng36 (pg/ml)	171.1 (75.2)	225.5 (148.8)	130.0 (80.9)
SNAP-25tot (pM)	49.2 (16.7)	71.4 (27.9)	53.5 (11.7)
SNAP-25aa40 (pM)	8.2 (3.4)	14.0 (6.3)	7.9 (2.3)
Synaptotagmin-1 (pM)	197.1 (78.9)	287.7 (156.0)	238.3 (71.4)

**Table 3:** Adjusted mean differences and 95% confidence intervals in CSF protein concentrations of the five synaptic measures between biomarker groups.

Group comparison	Ng22 (pg/ml)	Ng36 (pg/ml)	SNAP-25tot (pM)	SNAP-25aa40 (pM)	Synaptotagmin-1 (pM)
FTD vs AD biomarker group	*− 117.7 (− 193.8, − 54.2)*	− 54.0 (− 124.0, 10.3)	*− 21.9 (− 35.6, − 8.0)*	*− 5.7 (− 8.7, − 2.6)*	*− 88.6 (− 168.0, − 18.9)*
FTD biomarker vs control group	− 22.7 (− 78.4, 33.6)	41.7 (− 1.7, 84.0)	− 3.9 (− 11.4, 4.2)	0.5 (− 1.1, 2.3)	− 37.4 (− 80.4, 2.9)
AD biomarker vs control group	*95.0 (10.5, 169.6)*	*95.7 (20.7, 173.3)*	*18.0 (2.5, 32.4)*	*6.2 (2.9, 9.4)*	51.2 (− 30.3, 130.5)

Significant differences are shown in italics

Furthermore, concentrations of all of the synaptic markers were significantly increased in the AD biomarker group compared to the FTD biomarker group except for Ng36 where there was only a trend to higher levels. Concentrations in the FTD group were as follows: Ng22, 114.0 (117.5) pg/ml; Ng36, 171.1 (75.2) pg/ml; SNAP-25tot, 49.2 (16.7) pM; SNAP-25aa40, 8.2 (3.4) pM; and synaptotagmin-1, 197.1 (78.9) pM.

No significant differences were seen between the FTD biomarker group and the controls.

### Probable pathology stratification

**Fig. 2:**
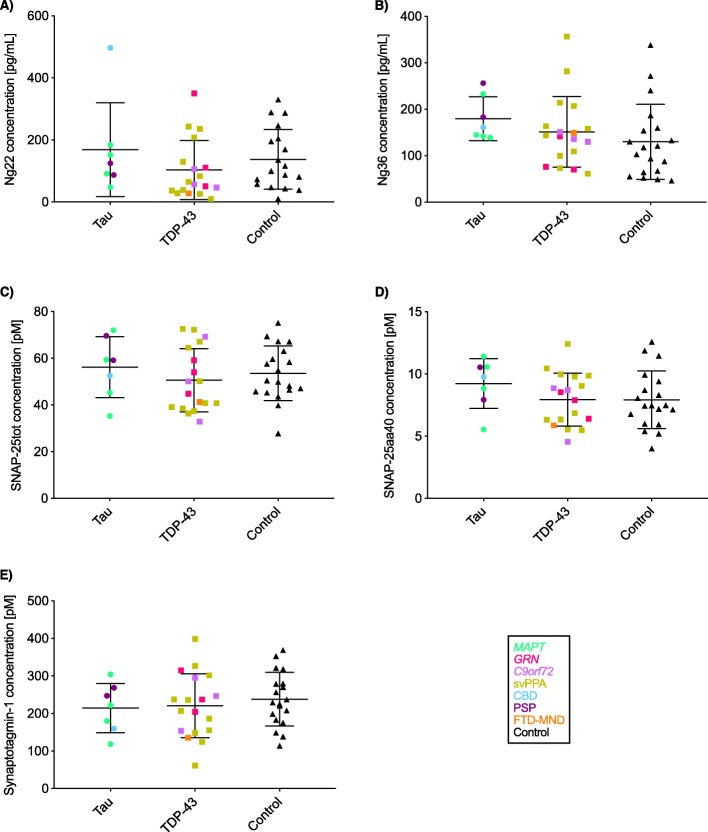
Concentrations for the five synaptic measures in patients with probable tau and TDP-43 pathology and controls: **a** Ng22, **b** Ng36, **c** SNAP-25tot, **d** SNAP-25aa40, and **e** synaptotagmin-1. Within each group, data points are coloured to represent the reason for allocation to each probable pathology group: green = *MAPT* mutation carrier, blue = pathologically confirmed CBD, dark purple = concomitant PSP, orange = concomitant MND, yellow = svPPA, pink = *GRN* mutation carrier, light purple = *C9orf72* mutation carrier, and black = controls

There were no significant differences in the comparison of the likely tau and TDP-43 groups with controls: Ng22, 169.0 (151.3) vs 103.1 (95.3) pg/ml; Ng36, 179.8 (47.2) vs 151.1 (75.9) pg/ml; SNAP-25tot, 56.2 (13.0) vs 50.6 (13.6) pM; SNAP-25aa40, 9.2 (2.0) vs 7.9 (2.1) pM; and synaptotagmin-1, 214.4 (65.3) vs 220.7 (85.0) pM (Fig. 2, Tables 4 and 5).

**Table 4:** CSF protein concentrations of each synaptic measure for those with likely tau and TDP-43 pathology

Synaptic measure	Tau (mean (SD))	TDP-43 (mean (SD))	Control (mean (SD))
Ng22 (pg/ml)	169.0 (151.3)	103.1 (95.3)	137.6 (95.9)
Ng36 (pg/ml)	179.8 (47.2)	151.1 (75.9)	130.0 (80.9)
SNAP-25tot (pM)	56.2 (13.0)	50.6 (13.6)	53.5 (11.7)
SNAP-25aa40 (pM)	9.2 (2.0)	7.9 (2.1)	7.9 (2.3)
Synaptotagmin-1 (pM)	214.4 (65.3)	220.7 (85.0)	238.3 (71.4)

**Table 5:** Adjusted mean differences and 95% confidence intervals in CSF protein concentrations of the five synaptic measures between the likely tau and TDP-43 groups, and controls

Group comparison	Ng22 (pg/ml)	Ng36 (pg/ml)	SNAP-25tot (pM)	SNAP-25aa40 (pM)	Synaptotagmin-1 (pM)
Tau vs TDP-43 group	65.9 (− 14.9, 246.4)	28.6 (− 22.7, 69.8)	5.6 (− 6.2, 15.2)	1.3 (− 0.4, 2.7)	− 6.4 (− 67.8, 47.8)
Tau vs control group	32.7 (− 45.3, 216.6)	51.5 (− 0.7, 101.7)	2.8 (− 8.8, 12.5)	1.4 (− 0.5, 3.0)	− 21.9 (− 79.5, 33.7)
TDP-43 vs control group	− 33.2 (− 97.6, 37.8)	23.0 (− 26.0, 78.5)	− 2.8 (− 10.9, 6.7)	0.2 (− 1.5, 1.6)	− 15.4 (− 67.7, 37.5)

No significant differences were seen

No significant differences in CSF synaptic protein concentrations were seen in the direct comparison between those with likely tau and TDP-43 pathology. However, there was a trend to an increased concentration of markers in the tau group compared to the TDP-43 group in all but synaptotagmin-1.

### Correlation with other fluid biomarkers

**Fig. 3:**
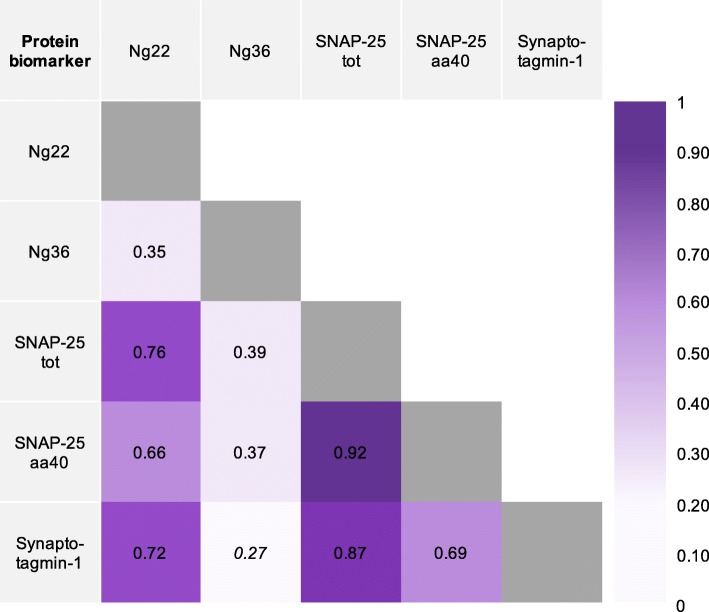
Correlation matrix displaying the Spearman correlation coefficients between CSF protein concentrations for each of the synaptic measures (Ng22, Ng36, SNAP-25tot, SNAP-25aa40, and synaptotagmin-1), for patients (i.e. controls not included). The gradient scale bar represents the strength of the correlation; the purple darkens as the correlation approaches a value of 1. Italicised coefficients represent biomarkers which were not significantly correlated (only Ng36 with synaptotagmin-1)

There were significant correlations between all synaptic marker concentrations except between Ng36 and synaptotagmin-1 (Fig. 3). In general, correlations were lower between Ng36 and the other markers.

All synaptic biomarkers, except Ng36, were significantly positively correlated with CSF total-tau concentrations in the FTD biomarker group (Ng22 *r* = 0.57; SNAP-25tot *r* = 0.71; SNAP-25aa40 *r* = 0.54; synaptotagmin-1 *r* = 0.68; all *p* < 0.0001) and AD biomarker group (Ng22 *r* = 0.86; SNAP-25tot *r* = 0.77; SNAP-25aa40 *r* = 0.73; synaptotagmin-1 *r* = 0.88; all *p* < 0.01). The concentrations of both SNAP-25 measures and synaptotagmin-1 were significantly positively correlated with CSF Aβ_42_ concentrations in the FTD biomarker group (SNAP-25tot *r* = 0.32; SNAP-25aa40 *r* = 0.35; synaptotagmin-1 *r* = 0.33; all *p* < 0.05) but not in the AD biomarker group.

## Discussion

We show that the synaptic proteins measured in this study are elevated in those with likely AD pathologically (i.e. atypical AD) but not in those with probable FTD pathology. Elevated levels of synaptic proteins in CSF are thought to reflect loss of synapses and therefore decreased concentration in the brain as proteins leak into the surrounding fluid. This pattern is well reported in AD, particularly for neurogranin but also for SNAP-25 and synaptotagmin-1 [2, 4, 5, 7–9, 12–14]. This study confirms those findings in a group with an atypical form of AD (mainly the logopenic variant of PPA).

It is unclear whether increased synaptic protein concentrations in CSF in AD but not FTD reflect the topography of volume loss, with anatomical targets of AD including areas rich in particular proteins, or instead the pathological process underlying AD [6, 9]. Neurogranin is mainly expressed in the cortex, amygdala, and hippocampus and enhances synaptic plasticity, critical for long term potentiation in the hippocampus and therefore learning and memory [2, 12, 20, 21]. Increased expression in regions affected in AD (i.e. hippocampus and parietal and temporal cortices) may explain the elevated levels of neurogranin in CSF from people with AD but not FTD, with greater synapse loss in these areas. However, in one previous study comparing neurogranin concentrations in typical and atypical AD, concentrations were higher in atypical AD (posterior cortical atrophy) than in controls, but higher still in amnestic AD [9]. Along with a lack of correlation between neurogranin concentrations and regional volumes, this suggests that the specificity of increased neurogranin to AD may be related to both the underlying pathogenesis of AD itself, as well as the anatomical involvement. Less is known about the anatomical distribution of SNAP-25 and synaptotagmin-1, although there appears to be relatively diffuse expression throughout the cortex [22–24], again suggestive that there may be some specific association with AD pathology rather than topography.

Synaptic markers were significantly correlated with total-tau concentration in both biomarker groups. Total-tau is significantly increased in the CSF of individuals with AD and to a lesser extent in those with FTD [25]. This correlation with total-tau has been reported elsewhere for each of the synaptic biomarkers, though not in FTD [2, 8, 13, 14], and is likely to represent the association of both total-tau and the synaptic biomarker concentration with the extent of neurodegeneration. Additionally, in the FTD biomarker group only, presynaptic SNAP-25 and synaptotagmin-1 concentrations correlated with Aβ_42_ concentration. In previous studies, the same correlation has been found with SNAP-25 and synaptotagmin-1 in controls but not in AD (where Aβ_42_ concentration is reduced) [13, 14]. It is unclear what this correlation represents, although a similar association with Aβ_42_ in an FTD group has been shown with other proteins [26].

In the present study, we also stratified participants by their probable frontotemporal lobar degeneration pathology to investigate any relationship with the underlying proteinopathy. While there were no significant differences in synaptic concentrations between those with probable tau and TDP-43 pathology, there was a trend to higher concentrations in the tau group across most synaptic biomarkers. Interestingly, one recent study revealed a positive correlation between CSF neurogranin concentration and postmortem tau neurofibrillary tangle pathology [12], suggesting a specific association with AD-tau pathology. Further work is therefore needed in larger groups with underlying non-AD tauopathies, as there may be specific associations of synaptic protein concentrations with particular disorders.

The lack of elevation of CSF synaptic protein concentrations in FTD biomarker group remains unexplained, particularly as synaptic dysfunction is well described in FTD [27]. As discussed above, this may represent the anatomical distribution of particular synaptic proteins measured (suggesting investigation of other markers that are expressed more widely across the cortex) or the specificity of their loss to the AD pathological process. It could also represent the extent of dysfunction (e.g. being of lower magnitude in FTD compared with AD, suggesting improvement in the sensitivity of current assays may be important). Another issue is that synaptic proteins are processed into fragments before being released into CSF [10, 21], and therefore, future assays should investigate alternative fragments of these proteins.

The limitations of this study include the relatively small sample size, especially of the FTD subgroups with probable tau and TDP-43 pathology. Additionally, the atypical AD group only included those with PPA clinically, particularly those with lvPPA, although patients with a bvFTD phenotype have also been reported to have underlying AD pathology on occasion [28]. Replication of these findings in a larger cohort would strengthen interpretation of our findings. Moreover, although we have reported a specificity of synaptic biomarkers to AD consistent with the existing literature, this study is the first to explore SNAP-25 and synaptotagmin-1 in FTD and verification is needed from independent cohorts. The cross-sectional design limits our evaluation of these synaptic proteins as prognostic biomarkers. Longitudinal measurements would enable investigations of CSF synaptic concentrations over time and whether these relate to cognitive performance and structural loss, which are important endpoints for clinical trials.

## Conclusions

In conclusion, none of the CSF synaptic proteins investigated here have been shown to be abnormal in those with likely FTD pathologically. Higher CSF synaptic protein concentrations of neurogranin, SNAP-25, and synaptotagmin-1 appear to be related to AD pathology. Biomarkers that track changes in pathophysiological events such as synapse dysfunction will be useful in the identification of novel therapeutic targets, or indeed markers to measure the effects of emerging therapies. The synaptic proteins presented here provide candidate biomarkers for disease pathogenesis in AD, though the search for synaptic biomarkers of FTD continues.

## Data Availability

The datasets analysed during the current study will be available from the corresponding author on reasonable request.

## Abbreviations

AD: Alzheimer’s disease
bvFTD: Behavioural variant frontotemporal dementia
CBD: Corticobasal degeneration
CI: Confidence interval
CSF: Cerebrospinal fluid
FTD: Frontotemporal dementia
lvPPA: Logopenic variant primary progressive aphasia
nfvPPA: Non-fluent variant primary progressive aphasia
Ng: Neurogranin
PPA: Primary progressive aphasia
PPA-NOS: Primary progressive aphasia-not otherwise specified
PSP: Progressive supranuclear palsy
SD: Standard deviation
SNAP-25: Synaptosomal-associated protein 25
svPPA: Semantic variant primary progressive aphasia
